# The association between bullying and early stages of suicidal ideation in late adolescents in Greece

**DOI:** 10.1186/1471-244X-11-22

**Published:** 2011-02-08

**Authors:** Petros Skapinakis, Stefanos Bellos, Tatiana Gkatsa, Konstantina Magklara, Glyn Lewis, Ricardo Araya, Stelios Stylianidis, Venetsanos Mavreas

**Affiliations:** 1 Department of Psychiatry, University of Ioannina, School of Medicine, Ioannina, Greece; 2 Academic Unit of Psychiatry, School of Social and Community Medicine, University of Bristol, Bristol, UK; 3 Department of Psychology, Panteion University of Social and Political Sciences, Athens, Greece

## Abstract

**Background:**

Bullying in schools has been associated with suicidal ideation but the confounding effect of psychiatric morbidity has not always been taken into account. Our main aim was to test the association between bullying behavior and early stages of suicidal ideation in a sample of Greek adolescents and to examine whether this is independent of the presence of psychiatric morbidity, including sub-threshold symptoms.

**Methods:**

5614 pupils 16-18 years old and attending 25 senior high schools were screened in the first phase and a stratified random sample of 2431 were selected for a detailed interview at the second phase. Psychiatric morbidity and suicidal ideation were assessed with the revised Clinical Interview Schedule (CIS-R) while bullying was assessed with the revised Olweus bully/victim questionnaire.

**Results:**

Victims of bullying behavior were more likely to express suicidal ideation. This association was particularly strong for those who were bullied on a weekly basis and it was independent of the presence of psychiatric morbidity (Odds Ratio: 7.78; 95% Confidence Interval: 3.05 - 19.90). In contrast, being a perpetrator ("bullying others") was not associated with this type of ideation after adjustment. These findings were similar in both boys and girls, although the population impact of victimization in the prevalence of suicidal ideation was potentially higher for boys.

**Conclusions:**

The strong cross-sectional association between frequent victimization and suicidal ideation in late adolescence offers an opportunity for identifying pupils in the school setting that are in a higher risk for exhibiting suicidal ideation.

## Background

Bulling is a specific form of aggression commonly reported among adolescents especially in the school setting [1-4]. Bulling in adolescence has been associated with general psychological distress or specific psychiatric disorders [5-10] and is considered to be a risk factor for the development of common mental disorders later in adulthood [11,12].

Of particular importance is the reported association between bullying and suicidal ideation [13-19] since suicide is a leading cause of mortality in adolescents which is potentially preventable [20]. Previous studies have established strong associations with suicidal ideation, mainly for the victims of bullying behavior. Interpretation of this association however is quite difficult and several factors should be taken into account before any firm conclusions about causality can be reached. Perhaps the most important issue is the confounding effect of psychiatric morbidity which is quite prevalent in adolescence and is associated with both bullying behavior [5-8,10] and suicidal ideation [21,22]. Despite this, from the previous 18 studies of the association between bullying and suicidal ideation (most are reviewed by Kim et al. 2009) [19] only six adjusted for the presence of psychiatric morbidity [13,17,19,23-25] and two of them found no association after adjustment [23,24]. In addition, most of these studies have assessed psychiatric conditions in a rather crude way using simple self-completed questionnaires. It is likely that a more detailed assessment of psychiatric morbidity could explain part of the residual confounding and could further reduce the reported associations between bullying and suicidal ideation. It is noted that confounding is an important issue irrespective of the study design and could influence the results of both cross-sectional and longitudinal studies.

Apart from confounding, an independent association between bullying and suicidal ideation would be further supported if there was evidence of a dose-response relationship whereby an increase in the intensity (either frequency or severity) of bullying would lead to greater reporting of suicidal ideation. One study that examined this issue failed to find consistently such a relationship [19]. In addition, there is some controversy in the literature about the relative associations of the different types of bullying behavior with suicidal ideation: most studies support that victims are more likely to report suicidal thoughts compared to perpetrators, while other studies have found that those who are both victims and perpetrators have the highest risk [13-15,17,19,25,26]. The latter implies an interaction between victims and perpetrators but most studies reporting this higher risk did not formally test for statistical interaction with appropriate methods.

Greece has one of the lowest suicide rates in the world [27] and this makes it interesting from an epidemiological perspective since establishment of an association between bullying and suicidal ideation in such an area may be less likely the result of unmeasured confounding factors. The present study used responses to the question "in the past week have you felt that life isn't worth living" [28] which is considered to be the first stage of the spectrum of suicidal ideation [28-30]. Our main aim was to test the association between bullying behavior and early stages of suicidal ideation in a sample of Greek adolescents and to examine whether this is independent of the presence of psychiatric morbidity assessed by means of a detailed structured interview.

## Methods

### Description of the data set and design of the study

The data reported here are coming from the Epirus School Project [31]. This was a cross-sectional survey carried out in selected upper secondary schools in Greece with the aim to investigate the prevalence and associations of common mental disorders in late adolescence.

### Sampling of Schools and Pupils

Upper secondary schools in Greece are either Senior High Schools (Lycea) or Technical Vocational Schools but 75% of students attend the first. In the current study only Senior High Schools were selected while Technical Vocational Schools will be included in a separate future survey. Approximately 75000 students attended 1193 Senior High Schools at the time of the design of the study. Schools were selected according to the following rules: a) all senior high schools of the major cities in the North-Western Part of Greece (Regions of Epirus and Aetoloakarnania) due to the proximity with the University of Ioannina, b) all senior high schools in one randomly selected district of the Athens Greater Area (the district of Kallithea was selected), c) all senior high schools of one island in the Aegean Sea (the island of Paros was conveniently selected).

All students in the selected schools were invited to participate in the study. Written consent for participation was actively obtained from both the students and their parents. Ethical approval for the study was also obtained by the Ministry of Education.

### Design of the study and data collection procedure

The study used a two-phase design [32]. In the first phase, all consenting students (N = 5614) were administered a brief screening instrument (see next section) in the classroom and then students were invited for the second phase using a stratified random sampling procedure according to the scores on the screening questionnaire: 100% of those scoring high on the screening instrument (>75^th ^percentile), 30% of those scoring in the middle and 10% of those scoring low (<25^th ^percentile). The second phase (N = 2431) consisted of the computerized version of a fully-structured psychiatric interview (see next section) and was carried out in the computer laboratories of the schools. The main fieldwork took place between January 2007 and April 2008.

### Assessment of Psychiatric Morbidity

Psychiatric symptoms were assessed with the revised clinical interview schedule (CIS-R), a fully structured psychiatric interview designed to be used by trained lay interviewers [33]. The CIS-R was the main instrument used in the national psychiatric morbidity surveys in the UK [34,35] and has been used in several other similar surveys around the world [36,37]. A computerized version has also been developed and found to be comparable with the regular interview [38]. The CIS-R was originally designed to assess symptoms in participants above 16 years old but has been previously used in teenagers above 14 years old in Australia [8,39].

The CIS-R assesses the presence and severity of 14 different common psychological symptoms (somatic symptoms, fatigue, concentration/memory problems, sleep problems, irritability, worry about physical health, depression, depressive ideas, worry, free-floating anxiety, phobias, panic, compulsions and obsessions). Two screening questions in each section ask about the presence of the symptom during the past month and then there is a more detailed assessment of the presence, frequency, duration, and severity of the symptom during the past seven days. Each symptom section is scored from 0 to 4 (except depressive ideas from 0 to 5) and a score of 2 or more denotes a clinically significant symptom and a total score of 18 or more indicates a clinical significant case [33]. Additional questions enable the application of the ICD-10 research diagnostic criteria using specially developed computerized algorithms [35].

For screening purposes in the first phase of the study we used the screening questions of the several symptom sections of the CIS-R. The full interview was given to those selected for the second phase (N = 2431)

The Greek version of the CIS-R has been validated and its psychometric properties have been published elsewhere [40]. The Cronbach's alpha for each symptom dimension ranged from 0.84 to 0.87 with an overall alpha for CIS-R of 0.86. A test-retest reliability of the CIS-R has been calculated in a subset of the present data set (two schools of the city of Ioannina with an interval between assessments of two weeks) and was found to be 0.84 [31]. For the purposes of the present study psychiatric morbidity can be assessed either in a dimensional way, using the total score on the CIS-R (by adding-up all 14 symptom dimensions), or in a categorical form using diagnostic categories. We have selected to use the total score in our analyses because in that way we are able to adjust for the full spectrum of psychiatric morbidity including sub-threshold forms of illness.

### Assessment of Suicidal Ideation

Suicidal ideation is commonly assessed by a set of questions of increasing severity that aim to investigate the full spectrum of suicidal thoughts and/or behaviour. The CIS-R starts by asking the following question about "tiredness of life": "in the past week have you felt that life isn't worth living?". Participants who reply positively are then asked the subsequent questions about death wishes ("have you wished that you were dead?") and actual suicide thoughts ("have you thought of taking your life even if you would not really do it?"). In the context of the present study we selected to ask all participants the first question only, without investigating further the more severe spectrum of suicidal ideation. We did this for the following reasons: a) our sample was not clinical and consisted of generally healthy adolescents attending secondary schools. We anticipated that the more severe spectrum of suicidal ideation would be relatively rare in this population and the statistical analysis would have been underpowered; b) there seems to be a continuum between less severe forms of suicidal ideation such as "tiredness of life" (as assessed by the "life isn't worth living" question) and the more severe forms of death wishes or actual suicidal ideas and there is not any clear cut-off to distinguish between these three groups [28]. Previous studies have shown that the pattern of associations with sociodemographic factors and psychiatric morbidity is the same between these groups and any observed differences are of a quantitative rather than a qualitative nature [28,41]; c) inclusion of the more severe forms of suicidal ideation in our survey would make necessary the implementation of an intervention for those pupils that would admit actual ideas of harming themselves. Such an intervention was not feasible for half of the schools that we planned to include in the study, therefore we preferred to exclude these questions in order to include a larger sample of schools and pupils. For all of the above reasons we opted for excluding these questions.

Participants could select three possible answers to the question of whether they were thinking that life was not worth living in the past week: "no", "yes sometimes", "yes all the time". As this question is the least severe form of the spectrum of suicidal ideation, we classified students as having suicidal ideation if they selected the third answer "all the time". All other students were classified into the "no/uncertain" category.

### Assessment of Bulling Behavior

Involvement in bullying either as a perpetrator (bully others) or as a victim (being bullied by others) was investigated in the second phase of the study using two questions taken from the revised Olweus Bully/Victim Questionnaire [42] which was also used in a WHO youth health study [43]. An introductory sentence defined bullying as follows:

''The next questions are about bullying. We say a pupil is being bullied when another pupil, or a group of pupils, says or does nasty and unpleasant things to him or her. It is also bullying when a pupil is teased repeatedly in a way he or she doesn't like. But it is not bullying when two pupils of about the same strength quarrel or fight.''

Thereafter the respondents were asked how frequently they had been bullied or they had bullied others, during the last 2 months in school. The possible answers were: "many times a week", "about once a week", "2 or 3 times per month", "1 or 2 times during the last 2 months" and "not at all". Based on these responses we classified participants into the following groups: a) Being a perpetrator ("bullying others") versus not being a perpetrator (reference category); b) being a victim versus not being a victim (reference category). We should like to note that this grouping allows the comorbidity between the two states, i.e. a perpetrator may also be a victim or vice versa. Other studies have used pure states ("pure" victims, "pure" perpetrators and both victim and perpetrators) but in our study we allowed comorbidity to investigate more formally whether there is statistical interaction between victims and perpetrators.

If the participant had been involved in this behavior at least once a week, this was classified as "frequent" bullying or victimization respectively, whereas all other instances were classified as " less frequent" bullying or victimization. Although this categorization is a bit arbitrary, it has been used in the past in other papers [9,10]. Bullying is considered to be a continuous process and including in the "bullying category" those pupils who had been involved once or twice during the past two months may not be universally accepted. However, we included those pupils in our definition, first to increase the statistical power of our study and second because empirically those pupils wee more similar regarding their association with psychiatric morbidity to the pupils with higher frequency bullying.

### Sociodemographic Variables

Information about several sociodemographic variables were obtained from the students in the first phase of the study (own age, parent's age, gender, parent's marital status, number of brothers and sisters, mother's educational status, father's educational status, mother's employment status, father's employment status). Students were also asked to subjectively rate their academic performance in school on a 4-point scale (excellent, very good, good, fair) and their relationship with mother and father (excellent, very good, good, fair, bad). In addition we asked students to subjectively assess their family's financial condition by asking them whether their family was having any financial difficulties (measured on a 4-point scale: no, few, some, a lot).

### Statistical Analysis

All analyses were performed with STATA/SE 9.2 (StataCorp, College Station, Texas). To take into account the potential effect of clustering of our data (since adolescents were nested into 25 schools) we first carried out a two-level logistic model (level 1: individuals, level 2: schools) in Stata using the gllamm command [44]. We also performed the models with the survey commands of Stata (svylogit) using school as the stratum. Results were very similar with both models and therefore in the paper we present the results using the survey commands because their use is more widespread in the literature. It should be noted that the effect of schools was negligible with an intraclass correlation coefficient close to zero (<0.08). In all analyses we have used probability weights to take into account the stratified random sampling procedure.

Adjusted population-attributable risk fractions (PAFs) and their 95% CIs were calculated from the final multivariable logistic regression model by using the *aflogit *procedure in Stata [45].

## Results

### Description of the sample

Overall 5,614 students took part in phase 1 of the study (55% girls, 41% 10^th ^grade, 28% 12^th ^grade) while in phase 2 we interviewed 2,431 students (59% girls, 39% 10^th ^grade, 29% 12^th ^grade). A detailed table of the sociodemographic characteristics of the whole sample in both phases of the study is given in additional file 1 - Table A1. Due to the stratified sampling procedure there were more female than male students in the second phase.

### Prevalence of Bullying/Victimisation and suicidal ideation

The prevalence of bullying/victimization by gender is shown in Table 1. It can be seen that being a perpetrator (but not a victim) was much more common among boys than girls (p < 0.001).

**Table 1:** Prevalence of bullying-related behaviours in 2431 Greek adolescents 16-18 years old

	Male	Female	Total
	N* (%)	N* (%)	N* (%)
«Bullied by others» - Victims			
Not at all	836 (87.2%)	1238 (89.3%)	2074 (88.2%)
Less frequent victimization (Less than weekly)	130 (11.3%)	173 (9.4%)	303 (10.4%)
Frequent victimization (Weekly)	22 (1.5%)	28 (1.3%)	50 (1.4%)
p** = 0.4103			2427 (100%)

«Bullying others» - Perpetrators			
Not at all	694 (72.4%)	1274 (89.1%)	1968 (80.7%)
Less frequent bullying others (Less than weekly)	240 (22.8%)	151(10.1%)	391 (16.5%)
Frequent bullying others (Weekly)	54 (4.8%)	14 (0.7%)	68 (2.8%)
p** < 0.001			2427 (100%)

* Actual number of observations; percentages in comparison are weighted to take into account the stratified random sampling procedure; **p-values from chi-squared tests corrected for the survey design of the study

Table 2 shows the prevalence of suicidal ideation by gender and by bullying behaviours. Thoughts that life is not worth living were reported more often from girls (5.1% vs. 2.4% for boys, p < 0.001). An increase in the frequency of victimization was associated with a higher prevalence of suicidal ideation (from 2.9% in not victimized students to 6.8% in less than weekly and 30.4% in weekly victimization, p < 0.001) while this was much weaker in students that bullied others (p = 0.09).

**Table 2:** Prevalence of suicidal ideation by bullying behaviour and gender in 2,431 Greek adolescents 16-18 years old

Gender	Suicidal ideation N (%)*
Male	46 (2.4%)
Female	108 (5.1%)
Total	154 (3.7%)
	p-value** <0.001

**Bullying Behaviors**	

**"Bullied by others" - Victims**	
No	110 (2.9%)
Yes, less frequent victimization	26 (6.8%)
Yes, frequent victimization	18 (30.4%)
	p-value** <0.001

**"Bully others" - Perpetrators**	
No	115 (3.3%)
Yes, less frequent bullying	32 (4.9%)
Yes, frequent bullying	7 (7%)
	p-value**= 0.090

* Actual number of observations; percentages in comparison are weighted to take into account the stratified random sampling procedure

**p-values from chi-squared tests corrected for the survey design of the study

### Logistic Regression analysis

Table 3 presents odds ratios and their 95% confidence intervals for the association between suicidal ideation and bullying behaviours. We present four models of increasing complexity: sex & age adjusted (model 1), additional adjustment for sociodemographic factors (model 2), additional adjustment for psychiatric morbidity (model 3), and finally additional adjustment for the concurrent presence of the opposite bullying behaviour (model 4). We also tested whether there was an interaction between victims and perpetrators by including an interaction term in the final model (victims*perpetrators). The likelihood ratio test however was not significant (= 1.01 on 2 degrees of freedom, p = 0.60) and since there was no evidence of interaction we present the simpler model with the main effects only.

**Table 3:** Adjusted Odds Ratios of suicidal ideation for different frequencies of victimization and perpetration in a sample of 2431 Greek Adolescents aged 16-18 years old

Odds Ratios (95% CI) of reporting Suicidal ideation				
	**Model 1:**	**Model 2:**	**Model 3:**	**Model 4:**
	adjusted for sex and age	Model 1 + socioeconomic and family factors*	Model 2 + psychiatric morbidity**	Model 3 + being simultaneously a bully**^† ^**or victim**^‡^**

**«Bullied by others» - Victims**				

**No**	1.00	1.00	1.00	1.00
**Yes, all frequencies**	3.72 (2.40 - 5.74)	3.43 (2.15 - 5.49)	2.03 (1.20 - 3.44)	1.94 (1.12 - 3.34)
*Yes, less frequent victimization*	2.55 (1.52 - 4.30)	2.38 (1.34 - 4.24)	1.42 (0.75 - 2.68)	1.34 (0.69 - 2.60)
*Yes, frequent victimization*	15.64 (7.33 - 33.35)	14.19 (6.58 - 30.59)	8.04 (3.14 - 20.62)	7.78 (3.05-19.90)

**«Bully others» - Perpetrators**				

**No**	1.00	1.00	1.00	1.00
**Yes, all frequencies**	2.11 (1.18 - 3.20)	1.72 (1.03 - 2.87)	1.50 (0.89 - 2.54)	1.35 (0.77 - 2.35)
*Yes, less frequent bullying*	1.94 (1.18 - 3.20)	1.70 (0.98 - 2.94)	1.54 (0.88 - 2.72)	1.39 (0.77 - 2.53)
*Yes, frequent bullying*	3.40 (1.27 - 9.07)	1.87 (0.75 - 4.66)	1.33 (0.58 - 3.03)	1.13 (0.47-2.72)

* age, gender, academic performance in school, parents' marital status, educational level and employment type of parents, type of relationship with parents, financial difficulties of the family; ** total score on the revised Clinical Interview Schedule (CIS-R); **^† ^**for the analysis of victims; **^‡ ^**for the analysis of perpetrators

**Figure 1:**
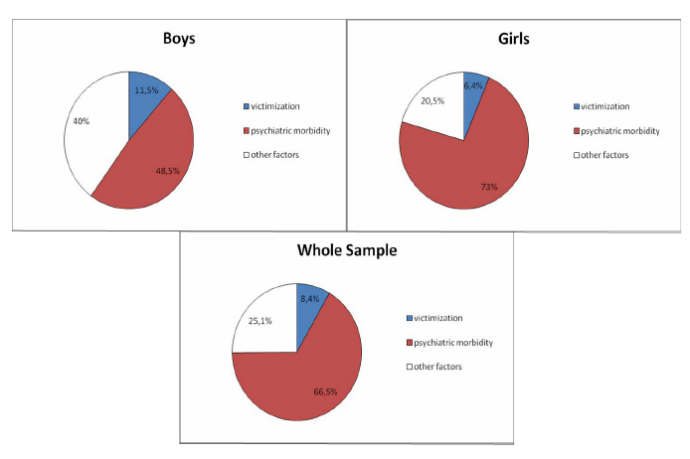
**Adjusted population attributable fractions for suicidal ideation apportioned to frequent victimization (being bullied weekly) and psychiatric morbidity in 2431 adolescents in Greece**.

Regarding victimisation, a robust significant association is noted in all models with evidence of a dose-response relationship in the less complex models. In models 3 and 4, the less frequent victimization category is no longer significant due to the inclusion of the psychiatric morbidity variable that acts as a confounder. Frequent victimization however is independently and strongly associated with suicidal ideation in all models. Subgroup analyses by sex showed that frequent victimization was strongly associated with suicidal ideation in both boys and girls in the fully adjusted model 4 (OR = 7.64 [95% CI: 2.18 - 26.78] for boys vs. 7.93 [1.88 - 33.35] for girls). However there was a non-significant trend for an association between low-frequency victimization and suicidal ideation in boys (p = 0.08) which was absent in girls (p = 0.97). To obtain an estimate of the population impact of frequent victimization in predicting suicidal ideation we calculated adjusted population attributable fractions (PAF) from the final model 4 (Figure 1). The adjusted PAF in the whole sample for frequent victimization was 8.4% (95% CI 4.4% - 12.2%). For comparison, the corresponding PAF for those with a high score on the psychiatric interview (CIS-R > = 18) was 66.5% (55.2% - 75.0%). The PAF for frequent victimization in boys was higher compared to girls.

Regarding the group of perpetrators ("bullying others"), an apparent association with suicidal ideation that was evident in the less complex models became non significant after adjustment for psychiatric morbidity. In the fully adjusted model, bullying others was not associated with suicidal ideation. A subgroup analysis by sex showed that this was true for both boys and girls.

## Discussion

### Main findings

In this cross-sectional study of late adolescents in Greece, a European country with low suicide rates, we found that victims of bullying behavior were more likely to express that "life was not worth living", an idea that is conceived to be part of the spectrum of suicidal ideation. This association was particularly strong for those who were bullied on a weekly basis and it was independent of the presence of psychiatric morbidity, assessed by means of a very detailed structured interview, and a wide range of other socioeconomic or family-related variables. In contrast, being a perpetrator ("bullying others") was not associated with this type of ideation after adjustment. These findings were similar in both boys and girls, although the population impact of victimization in the prevalence of suicidal ideation was potentially higher for boys.

### Limitations

These findings should be interpreted in the context of the following limitations: a) the cross sectional nature of the study does not allow us to study the temporal association between suicidal ideation and bullying behaviors; b) suicidal ideation was crudely assessed with a simple question on "tiredness of life". We did not ask further questions on more severe forms of suicidal ideation (death wishes or actual ideas of harming oneself) for the reasons we have explained in detail in the methods section. Therefore, our results cannot be generalized to more severe forms of suicidal ideation. It is possible, for example, that the role of depression or other psychiatric disorders in more severe forms of suicidal ideation could be more important; c) our sample did not include adolescents attending technical vocational schools (approximately 25% of the adolescents of this age attend this type of school). Our results, however, are applicable to the remaining adolescents continuing their secondary education in Greece.

### Comparison with previous studies and interpretation of the findings

We are not aware of other studies of the association between bullying and suicidal ideation in Greece and therefore we will base our discussion on studies carried out in other countries. It should be noted however that despite the considerable variation in rates of suicidal ideation or behaviors across countries, risk factors are often similar [46,47].

The association between bullying behaviors and suicidal ideation is a complex one and at least three issues need careful examination: a) psychiatric conditions are expected to have a strong confounding effect that needs to be taken into account; b) victims and perpetrators (bullying others) may differ in their suicidal risk; c) the longitudinal relationship between bullying and suicidal risk could go in both directions or even bullying and suicidal behaviors may follow parallel trajectories over time [48,49].

Previous studies of the association between bullying and suicide behaviors have not consistently adjusted for psychiatric disorders as has already been noticed earlier in this paper [13,17,19,23-25]. This is a serious limitation of the literature given the strong association between bullying and psychiatric disorders on the one hand and suicidal ideation and psychiatric disorders on the other [50]. In our study we confirmed the confounding effect of psychiatric morbidity especially for the "bullying others" category. After adjustment, pupils who bullied others did no longer showed an increased risk for reporting suicidal ideation. It is worth noting that in our study we used the total score on the psychiatric morbidity interview and not a binary category of psychiatric disorder versus non-disorder. By doing this we also adjusted for sub-threshold symptoms that may play an important role. In contrast, most previous studies have used binary categories and therefore there may be residual confounding not taken into account.

Differences in the way the confounding effect of psychiatric morbidity has been controlled for may explain the reported inconsistencies of the literature regarding the specific association of victims and perpetrators with suicidal ideation. Victimization has been consistently associated with suicidal ideation [19]. It is uncertain though whether perpetrators are at an increased risk. It is worth noting that most of the previous studies have coded pure bullying behaviors into different variables and a third category has been assigned to those who show both behaviors at the same time (both victims and perpetrators). However, this is justified only in the presence of statistically significant interactions [51], and most of the studies did not carry out such a test. In the Kim et al. (2005) [17] study for example it is reported that pure victims but not pure perpetrators were significantly more likely to show suicidal/self injurious behavior over the past 6 months (odds ratios 1.69 versus 1.16). The authors also report that those who were both victims and perpetrators were also significantly more likely to show such behavior but the magnitude of the odds ratio (1.85) is commensurate with adding the main effects of pure victims and pure perpetrators with no indication of a statistically significant interaction effect. The longitudinal study of Brunstein-Klomek et al. [25] also failed to find a significant effect for perpetrators in predicting future suicide behavior after adjustment for psychiatric disorders. In contrast, victimization in girls was found significant and a similar non-significant trend was reported for boys. From the previous studies only Kaltiala-Heino et al. [13] in Finland have reported main effects for perpetrators that was higher than that of victims after adjustment for depression. However the authors did not adjust for other psychiatric symptoms (e.g. conduct problems) that are known to act as confounders in the association between suicidal ideation and bullying others [24,25]. In our study the interaction term between victims and perpetrators was not significant and therefore we present the simpler models. Our results show that victims of bullying behavior are at a higher risk for reporting suicidal ideation. This association was especially high in those who were frequently bullied showing some evidence of a dose-response association. We did not find an association between bullying others and suicidal ideation after adjustment and therefore our findings support the notion that the two groups (victims or perpetrators) may differ regarding their specific association with suicidal ideation [25]. It is worth noting that most of the previous studies including our own have not collected data on the third category of pupils who observe the bullying-related behaviour ("bystanders"). In a recent study in schools in North of England [52] there was some evidence that this group may also be at an increased risk for suicidal ideation and therefore future studies should aim to explore further whether there is any mediating effect of this type of observational behaviour in the association between victimization/bullying and suicidal ideation.

Our cross-sectional study does not allow investigating the temporal sequence of victimization and suicide ideation, although the few longitudinal studies show that the bullying behaviors usually precede suicidal ideation [8]. Apart from the temporal sequence a strong cross-sectional association could also imply that bullying and suicidal ideation may follow a parallel trajectory over time. It is worth noting that in the Kim et al. (2009) [19] longitudinal study there was some evidence that suicidal ideation or behaviors at follow-up were more strongly associated with incident victimization compared to baseline only and this supports the idea that victimization and suicidal ideation may show a synchrony of change.

## Conclusions

The strong cross-sectional association between frequent victimization and suicidal ideation in late adolescence offers an opportunity for identifying pupils in the school setting that are at a higher risk for exhibiting suicidal ideation or behaviors. Victims of bullying behavior in the school setting are relatively easy to identify and specially designed anti-bullying programs in schools [53] can also help in the more efficient detection of frequently bullied pupils. Victims of bullying behavior should have easy access to professional help. If depression or other mental health problem is detected treatment should be readily available. Although it is still uncertain whether victimization is a marker or a genuine risk factor of suicidal ideation or behavior, our calculation of population attributable fractions shows that if it is a risk factor one can expect a small but clinically important reduction in suicidal ideation if bullying could be prevented in the school setting in Greece. It is likely that in other cultures with higher suicidal rate this may be even more important. Future longitudinal studies should also investigate whether reductions in bullying behaviors are associated with reductions in suicidal ideation or behaviors and the possibility of conducting randomized controlled trials on this issue should be further explored.

## Competing interests

The authors declare that they have no competing interests.

## Authors' contributions

PS was responsible for the conception and design of the study, helped in data collection, contributed to the statistical analysis and drafted the manuscript. SB helped in data collection, in the statistical analysis, in the writing of the manuscript and interpretation of the results. TG helped in data collection and interpretation of the results. KM helped in data collection, contributed to the statistical analyses and interpretation of the results. GL made critical comments and helped in the interpretation of the results. RA made critical comments and helped in the interpretation of the results. SS helped in data collection and made critical comments that helped in the interpretation of the results. VM helped in obtaining funding for the study, in the writing of the paper and interpretation of the results. All authors read and approved the final manuscript.

## Pre-publication history

The pre-publication history for this paper can be accessed here:

http://www.biomedcentral.com/1471-244X/11/22/prepub

## Supplementary Material

Additional file 1 **Table A1**. Click here for file

## References

[B1] Olweus D Bullying at school: What we know and what we can do 1993 Oxford Blackwell

[B2] Olweus D Smith PK, Morita Y, Junger-Tas J, Olweus D, Catalano R, Slee P Sweden The Nature of School Bullying: A Cross-national Perspective 1999 New York, NY: Routledge 7 27

[B3] Smith-Khuri E Iachan R Scheidt PC Overpeck MD Gabhainn SN Pickett W Harel Y A cross-national study of violence-related behaviors in adolescents Arch Pediatr Adolesc Med 2004 158 539 44 10.1001/archpedi.158.6.539 15184216

[B4] Due P Holstein BE Lynch J Diderichsen F Gabhain SN Scheidt P Currie C Health Behaviour in School-Aged Children Bullying Working Group. Bullying and symptoms among school-aged children: international comparative cross sectional study in 28 countries Eur J Public Health 2005 15 128 32 10.1093/eurpub/cki105 15755782

[B5] Salmon G James A Smith DM Bullying in schools: self reported anxiety, depression, and self esteem in secondary school children BMJ 1998 317 924 5 9756812 10.1136/bmj.317.7163.924 PMC28678

[B6] Forero R McLellan L Rissel C Bauman A Bullying behaviour and psychosocial health among school students in New South Wales, Australia: cross sectional survey BMJ 1999 319 344 8 10435953 10.1136/bmj.319.7206.344 PMC28186

[B7] Kaltiala-Heino R Rimpelä M Rantanen P Rimpelä A Bullying at school--an indicator of adolescents at risk for mental disorders J Adolesc 2000 23 661 74 10.1006/jado.2000.0351 11161331

[B8] Bond L Carlin JB Thomas L Rubin K Patton G Does bullying cause emotional problems? A prospective study of young teenagers BMJ 2001 323 480 4 10.1136/bmj.323.7311.480 11532838 PMC48131

[B9] Nansel TR Overpeck M Pilla RS Ruan WJ Simons-Morton B Scheidt P Bullying behaviors among US youth: prevalence and association with psychosocial adjustment JAMA 2001 285 2094 100 10.1001/jama.285.16.2094 11311098 PMC2435211

[B10] Nansel TR Craig W Overpeck MD Saluja G Ruan WJ Health Behaviour in School-aged Children Bullying Analyses Working Group. Cross-national consistency in the relationship between bullying behaviors and psychosocial adjustment Arch Pediatr Adolesc Med 2004 158 730 6 10.1001/archpedi.158.8.730 15289243 PMC2556236

[B11] Roth DA Coles ME Heimberg RG The relationship between memories for childhood teasing and anxiety and depression in adulthood J Anxiety Disord 2002 16 149 64 10.1016/S0887-6185(01)00096-2 12194541

[B12] Sourander A Jensen P Rönning JA Niemelä S Helenius H Sillanmäki L Kumpulainen K Piha J Tamminen T Moilanen I Almqvist F What is the early adulthood outcome of boys who bully or are bullied in childhood? The Finnish "From a Boy to a Man" study Pediatrics 2007 120 397 404 10.1542/peds.2006-2704 17671067

[B13] Kaltiala-Heino R Rimpelä M Marttunen M Rimpelä A Rantanen P Bullying, depression, and suicidal ideation in Finnish adolescents: school survey BMJ 1999 319 348 51 10435954 10.1136/bmj.319.7206.348 PMC28187

[B14] Roland E Bullying, depressive symptoms and suicidal thoughts Educational research 2002 44 55 67 10.1080/00131880110107351

[B15] van der Wal MF de Wit CA Hirasing RA Psychosocial health among young victims and offenders of direct and indirect bullying Pediatrics 2003 111 1312 7 10.1542/peds.111.6.1312 12777546

[B16] Ivarsson T Broberg AG Arvidsson T Gillberg C Bullying in adolescence: psychiatric problems in victims and bullies as measured by the Youth Self Report (YSR) and the Depression Self-Rating Scale (DSRS) Nord J Psychiatry 2005 59 365 73 10.1080/08039480500227816 16757465

[B17] Kim YS Koh YJ Leventhal B School bullying and suicidal risk in Korean middle school students Pediatrics 2005 115 357 63 10.1542/peds.2004-0902 15687445

[B18] Klomek AB Marrocco F Kleinman M Schonfeld IS Gould MS Bullying, depression, and suicidality in adolescents J Am Acad Child Adolesc Psychiatry 2007 46 40 9 10.1097/01.chi.0000242237.84925.18 17195728

[B19] Kim YS Leventhal BL Koh YJ Boyce WT Bullying increased suicide risk: prospective study of Korean adolescents Arch Suicide Res 2009 13 15 30 10.1080/13811110802572098 19123106

[B20] Anderson RN Deaths: Leading causes for 2000 Natl Vital Stat Rep 2002 50 1 85 12355905

[B21] Beautrais AL Joyce PR Mulder RT Fergusson DM Deavoll BJ Nightingale SK Prevalence and comorbidity of mental disorders in persons making serious suicide attempts: a case-control study Am J Psychiatry 1996 153 1009 14 8678168 10.1176/ajp.153.8.1009

[B22] Pelkonen M Marttunen M Child and adolescent suicide: epidemiology, risk factors, and approaches to prevention Paediatr Drugs 2003 5 243 65 12662120 10.2165/00128072-200305040-00004

[B23] Park HS Schepp KG Jang EH Koo HY Predictors of suicidal ideation among high school students by gender in South Korea J Sch Health 2006 76 181 8 10.1111/j.1746-1561.2006.00092.x 16635202

[B24] Klomek AB Sourander A Kumpulainen K Piha J Tamminen T Moilanen I Almqvist F Gould MS Childhood bullying as a risk for later depression and suicidal ideation among Finnish males J Affect Disord 2008 109 47 55 10.1016/j.jad.2007.12.226 18221788

[B25] Klomek AB Sourander A Niemelä S Kumpulainen K Piha J Tamminen T Almqvist F Gould MS Childhood bullying behaviors as a risk for suicide attempts and completed suicides: a population-based birth cohort study J Am Acad Child Adolesc Psychiatry 2009 48 254 61 10.1097/CHI.0b013e318196b91f 19169159

[B26] Liang H Flisher AJ Lombard CJ Bullying, violence, and risk behavior in South African school students Child Abuse Negl 2007 31 161 71 10.1016/j.chiabu.2006.08.007 17313977

[B27] Chishti P Stone DH Corcoran P Williamson E Petridou E EUROSAVE Working Group Suicide mortality in the European Union Eur J Public Health 2003 13 108 14 10.1093/eurpub/13.2.108 12803408

[B28] Dennis M Baillon S Brugha T Lindesay J Stewart R Meltzer H The spectrum of suicidal ideation in Great Britain: comparisons across a 16-74 years age range Psychol Med 2007 37 795 805 10.1017/S0033291707000013 17288647

[B29] Goldney RD Wilson D Dal Grande E Fisher LJ McFarlane AC Suicidal ideation in a random community sample: attributable risk due to depression and psychosocial and traumatic events Aust N Z J Psychiatry 2000 34 98 106 10.1046/j.1440-1614.2000.00646.x 11185952

[B30] Lewinsohn P Rohde P Seeley J Adolescent Suicidal Ideation and Attempts: Prevalence, Risk Factors, and Clinical Implications Clinical Psychology: Science and Practice 1996 3 25 46 10.1111/j.1468-2850.1996.tb00056.x

[B31] Skapinakis P Bellos S Mihalis G Gkatsa T Mavreas V The epidemiology of common mental disorders in adolescents: The Epirus school project [abstract] Eur Psychiatry 2007 22 S331 10.1016/j.eurpsy.2007.01.1105

[B32] Dunn G Pickles A Tansella M Vazquez-Barquero JL Two-phase epidemiological surveys in psychiatric research Br J Psychiatry 1999 174 95 100 10.1192/bjp.174.2.95 10211161

[B33] Lewis G Pelosi AJ Araya RC Dunn G Measuring psychiatric disorder in the community: a standardized assessment for use by lay-interviewers Psychol Med 1992 22 465 486 10.1017/S0033291700030415 1615114

[B34] Jenkins R Bebbington P Brugha T Farrell M Gill B Lewis G Meltzer H Petticrew M The National Psychiatric Morbidity surveys of Great Britain--strategy and methods Psychol Med 1997 7 765 74 10.1017/S003329179700531X 9234455

[B35] Singleton N Bumpstead R O'Brien M Lee A Meltzer H Psychiatric morbidity among adults living in private households 2000 Int Rev Psychiatry 2003 15 65 73 10.1080/0954026021000045967 12745312

[B36] Botega NJ Pereira WA Bio MR Garcia Junior C Zomignani MA Psychiatric morbidity among medical in-patients: a standardized assessment (GHQ-12 and CIS-R) using 'lay' interviewers in a Brazilian hospital Soc Psychiatry Psychiatr Epidemiol 1995 30 127 31 10.1007/BF00802041 7624806

[B37] Araya R Rojas G Fritsch R Acuna J Lewis G Common mental disorders in Santiago, Chile: prevalence and socio-demographic correlates Br J Psychiatry 2001 178 228 33 10.1192/bjp.178.3.228 11230033

[B38] Lewis G Pelosi AJ Glover E Wilkinson G Stansfeld SA Williams P Shepherd M The development of a computerized assessment for minor psychiatric disorder Psychol Med 1988 18 737 45 10.1017/S0033291700008448 3054992

[B39] Patton GC Coffey C Posterino M Carlin JB Wolfe R Bowes G A computerised screening instrument for adolescent depression: population-based validation and application to a two-phase case-control study Soc Psychiatry Psychiatr Epidemiol 1999 34 166 72 10.1007/s001270050129 10327843

[B40] Skapinakis P Anagnostopoulos F Bellos S Magklara K Lewis G Mavreas V An empirical investigation of the structure of anxiety and depressive symptoms in late adolescence: cross-sectional study using the Greek version of the revised Clinical Interview Schedule Psychiatry Res 2010 in press http://doi:10.1016/j.psychres.2010.08.023 20846727 10.1016/j.psychres.2010.08.023

[B41] Thomas HV Crawford M Meltzer H Lewis G Thinking life is not worth living. A population survey of Great Britain Soc Psychiatry Psychiatr Epidemiol 2002 37 351 6 10.1007/s00127-002-0556-5 12195541

[B42] Olweus D The Revised Olweus Bully/Victim Questionnaire 1996 Bergen, Norway: University of Bergen

[B43] WHO Health and health behaviour among young people: health behaviour in school-aged children: a WHO cross-national study (HBSC): international report Copenhagen: Health Promotion and Investment for Health 2000 World Health Organization Regional Office for Europe

[B44] Rabe-Hesketh S Skrondal A Multilevel and longitudinal modelling using Stata 2008 College Station, TX: Stata Press

[B45] Brady AR Adjusted population attributable fractions from logistic regression (sbe21) Stata Technical Bulletin 1998 42 8 12

[B46] Bernal M Haro JM Bernert S Brugha T de Graaf R Bruffaerts R Lépine JP de Girolamo G Vilagut G Gasquet I Torres JV Kovess V Heider D Neeleman J Kessler R Alonso J ESEMED/MHEDEA Investigators Risk factors for suicidality in Europe: results from the ESEMED study J Affect Disord 2007 101 27 34 10.1016/j.jad.2006.09.018 17074395

[B47] Nock MK Borges G Bromet EJ Alonso J Angermeyer M Beautrais A Bruffaerts R Chiu WT de Girolamo G Gluzman S de Graaf R Gureje O Haro JM Huang Y Karam E Kessler RC Lepine JP Levinson D Medina-Mora ME Ono Y Posada-Villa J Williams D Cross-national prevalence and risk factors for suicidal ideation, plans and attempts Br J Psychiatry 2008 192 98 105 10.1192/bjp.bp.107.040113 18245022 PMC2259024

[B48] Judd LL Akiskal HS Zeller PJ Paulus M Leon AC Maser JD Endicott J Coryell W Kunovac JL Mueller TI Rice JP Keller MB Psychosocial disability during the long-term course of unipolar major depressive disorder Arch Gen Psychiatry 2000 57 375 80 10.1001/archpsyc.57.4.375 10768699

[B49] Ormel J Synchrony of change in depression and disability: what next? Arch Gen Psychiatry 2000 57 381 2 10.1001/archpsyc.57.4.381 10768700

[B50] Gould MS King R Greenwald S Fisher P Schwab-Stone M Kramer R Flisher AJ Goodman S Canino G Shaffer D Psychopathology associated with suicidal ideation and attempts among children and adolescents J Am Acad Child Adolesc Psychiatry 1998 37 915 23 10.1097/00004583-199809000-00011 9735611

[B51] Assmann SF Pocock SJ Enos LE Kasten LE Subgroup analysis and other (mis)uses of baseline data in clinical trials Lancet 2000 355 1064 9 10.1016/S0140-6736(00)02039-0 10744093

[B52] Rivers I Noret N Participant roles in bullying behavior and their association with thoughts of ending one's life Crisis 2010 31 143 48 20573608 10.1027/0227-5910/a000020

[B53] Mytton J DiGuiseppi C Gough D Taylor R Logan S School-based secondary prevention programmes for preventing violence Cochrane Database Syst Rev 2006 3 CD004606 16856051 10.1002/14651858.CD004606.pub2 PMC8842960

