# Supplementary material for: The association between bullying and early stages of suicidal ideation in late adolescents in Greece
Source: BMC Psychiatry. 2011 Feb 8;11:22. doi: 10.1186/1471-244X-11-22 (PMC3042930; doi:10.1186/1471-244X-11-22)
Supplement: Additional file 1 — Table A1. [file 1471-244X-11-22-S1.DOC]

# Additional File 1

**Table A**1. Basic Description of the Sample in the two phases of the study

| **Variable** | **Phase 1 (n=5614)** | **Phase 2 (N=2431)** |
| --- | --- | --- |
| **Gender**  Male  Female | 2530 (45%)  3084 (55%) | 989 (41%)  1442 (59%) |
| **Age**  ≤16  17  ≥18 | 2265 (41%)  1869 (33%)  1440 (26%) | 957 (40%)  825 (34%)  627 (26%) |
| **Grade**  10^th^ grade  11^th^ grade  12^th^ grade | 2281 (41%)  1772 (31%)  1561 (28%) | 942 (39%)  778 (32%)  711 (29%) |
| **Parent’s Family Status**  Married  Divorced / Separated  Widow  Missing | 5012 (89%)  394 (7%)  159 (3%)  49 (1%) | 2145 (88%)  178 (7.5%)  82 (3.5%)  26 (1%) |
| **Father’s Age** | 47.97 (5.14) | 47.8 (4.9) |
| **Mother’s Age** | 42.60 (4.7) | 427 (4.6) |
| **Number of brothers/sisters**  None  One  Two  Three or more | 446 (8%)  3087 (55%)  1313 (24%)  722 (13%) | 194 (8%)  1.407 (59,5%)  537 (23%)  225 (9,5%) |
| **Father’s Employment**  Employed – Public Sector  Employed – Private Sector  Self-employed  Unemployed  Retired  Other/Missing | 1828 (33%)  1183 (22%)  1949 (35.5%)  42 (0.5%)  290 (5%)  224 (4%) | 796 (33%)  511 (21%)  839 (34.5%)  17 (0.5%)  123 (5%)  145 (6%) |
| **Mother’s Employment**  Employed – Public Sector  Employed – Private Sector  Self-employed  Looks after House  Unemployed  Other/Missing | 1679 (30%)  1056 (19%)  741 (13%)  1720 (31%)  234 (4%)  184 (3%) | 681 (28%)  446 (18.5%)  347 (14%)  748 (31%)  109 (4.5%)  100 (4%) |
| **Father’s Educational Status**  Primary  Secondary Basic  Secondary Complete  Technological degree  University degree | 791 (14%)  849 (15%)  1589 (29%)  738 (13%)  1562 (28%) | 382 (16%)  344 (14%)  733 (30%)  309 (13%)  663 (27%) |
| **Mother’s Educational Status**  Primary  Secondary Basic  Secondary Complete  Technological degree  University degree | 743 (13%)  784 (14%)  2086 (37.5%)  584 (10.5%)  1385 (25%) | 338 (14%)  365 (15%)  915 (37.5%)  233 (9.5%)  580 (24%) |
| **Financial Difficulties**  None  Very little  Some  A lot | 1776 (32%)  3028 (54%)  675 (12%)  118 (2%) | 708 (29%)  1317 (54.5%)  328 (13.5%)  66 (3%) |
| **School Performance**  Excellent  Very Good  Good  Fair | 510 (9%)  1898 (34%)  2135 (38%)  1047 (19%) | 209 (8.5%)  751 (31%)  932 (38.5%)  526 (22%) |
